# Addressing a clinical challenge: guidelines for the diagnosis and treatment of leishmaniasis

**DOI:** 10.1186/s12916-017-0843-3

**Published:** 2017-04-07

**Authors:** Naomi E. Aronson

**Affiliations:** Division of Infectious Diseases, Uniformed Services University of the Health Sciences, 4301 Jones Bridge Road, Bethesda, MD 20814 USA

**Keywords:** Leishmaniasis, Clinical practice guidelines, Diagnosis, Treatment

## Abstract

Leishmaniasis is a chronic intracellular parasitic infection that travelers, immigrants, deployed military personnel, and refugees from endemic global areas acquire from the bite of infected sand flies and carry with them, including to non-endemic countries where leishmaniasis may be an unfamiliar illness to medical providers. This commentary discusses the first clinical practice guidelines produced by the Infectious Diseases Society of America and American Society of Tropical Medicine and Hygiene for the diagnosis and management of leishmaniasis, targeted for clinicians in North America.

## Background

Leishmaniasis, caused by various *Leishmania* parasites and transmitted by the bite of an infected sand fly vector, is on the rise [1]. An immunologically unprotected host who travels to any of the 88 countries with areas of endemic leishmaniasis is at risk of acquiring infection. Whether the inciting event be population displacement due to conflict or food insecurity, refugee relocation, eco-tourism, urbanization, or northward extension of the sand fly vector with global warming trends, the end result is that more patients with cases of leishmaniasis will present to healthcare systems in non-endemic Europe and North America. Some countries (e.g., India, Bangladesh, Nepal) are working toward eliminating visceral leishmaniasis (VL); however, VL epidemics occur in Sudan and South Sudan, and there are increased rates of VL cases in Brazil as a result of populations moving from rural to densely populated peri-urban regions. Recent epidemics of cutaneous leishmaniasis (CL) have been associated with an earthquake in Bam, Islamic Republic of Iran; with refugees and displaced persons in Kabul, Afghanistan; with the US military operations in Operation Iraqi Freedom 2003–2005; and as a consequence of the conflict in Syria.

The United Nations High Commissioner for Refugees reported that 65 million persons were displaced in 2015 and that three countries provided one half of the worlds’ refugees: Syria, Afghanistan, and Somalia [2]. In two of these countries, Syria and Afghanistan, leishmaniasis is endemic. The visible signs of CL (chronic skin sores often on exposed parts of the body) broadcast an obvious indication of emerging infection in unprotected populations. While the Syrian Ministry of Health reported a twofold increase in CL cases in 2013, some feel the number of CL cases could exceed 100,000 per year [3]. In a GeoSentinel surveillance study, 32% of adult Syrian refugees evaluated for migration-related illness had CL [4]. Increased CL rates were also reported in the neighboring countries of Jordan [5], Lebanon [6, 7], and Turkey [8, 9], primarily among Syrian refugee populations. Poor living conditions, disrupted healthcare infrastructure, vector control programs, inadequate sanitation, and mass migration from non-endemic regions of Syria through endemic areas all likely contribute to an increased disease burden of leishmaniasis in Syrian refugees.

An additional contributor to the rise of VL is decreased host susceptibility. Globally, this is most commonly caused by malnutrition, but in high-resourced countries this can also be caused by immunosuppression. Causative factors for the latter include HIV–*Leishmania* co-infection, initially recognized in Mediterranean intravenous drug users but now seen worldwide, and risks associated with biologic immunomodulating drugs, including post transplantation, and with the burgeoning use of tumor necrosis factor-alpha inhibitors [10, 11].

## Discussion

In 2007, the World Health Assembly passed a resolution on the control of leishmaniasis, raising awareness of these infections [12]. Subsequently, several major clinical guidelines have been published, but they were not targeted toward clinical management in North America [13]. In the USA, there is a dichotomous dilemma with the diagnosis and care of patients with leishmaniasis. Healthcare is well resourced, but leishmaniasis is not endemic and most providers may not recognize the typical signs and symptoms. Aside from histopathology, diagnostic testing is relegated to a few reference laboratories only. Most drugs used globally to treat leishmaniasis are not approved by the Food and Drug Administration for use in the USA and may not be easily available. In light of these challenges, detailed clinical practice guidelines for the diagnosis and management of leishmaniasis in North America were recently published [14, 15] after an inaugural collaboration between the Infectious Diseases Society of America (IDSA) and the American Society of Tropical Medicine and Hygiene (ASTMH).

The IDSA-ASTMH leishmaniasis clinical practice guidelines were structured by including questions that commonly arise in clinical practice. They provide practical tables, maps of endemic areas, clinical photographs, and contact details for reference diagnostic laboratories. These guidelines are detailed; they are organized into nine diagnostic questions (22 recommendations) and 26 treatment questions (57 recommendations) covering CL, mucosal leishmaniasis (ML), VL, and leishmaniasis management in the immunocompromised host and in special populations (young children, elderly persons, women who are pregnant or lactating, and persons with medical comorbidities). Only the executive summary is published in paper format, but each question has an associated evidence summary available online. The guidelines are extensively referenced (>500 references) and a supplemental appendix has a searchable document of controlled CL treatment trials, transparently allowing a provider to assess the quality of evidence, and allowing them to search by country of acquisition, *Leishmania* species, and type/route of treatment. Many of the guideline recommendations derive from observational trials and case reports, or consist of expert opinion. Recommendations are given based on a determination of the strength of the recommendation (strong or weak) and the quality of evidence (high, moderate, low, very low). In the guidelines, 14% of the diagnostic recommendations and 16% of the therapeutic recommendations were rated as weak; with regards the quality of evidence, diagnostic (32% moderate, 48% low, 19% very low) and therapeutic (4% high, 20% moderate, 49% low, and 27% very low) recommendations were skewed to lower-quality studies.

In the context of a majority of low/very low quality of evidence, applying GRADE (grading of recommendations, assessment, development, and evidence) methodology is challenging. Early on, the committee decided to address questions for clinicians, whether or not the published evidence was adequate. The guidelines took in excess of 5 years to complete and are lengthy as well as complex. For the North American audience, new areas of focus include managing immunocompromised hosts (not just HIV) and special populations, and a discussion of the new drug, miltefosine, and its current role in treatment. Local therapies are reviewed for treating uncomplicated leishmaniasis, such as Old World CL; these approaches have been underutilized in the USA. Some of the more controversial recommendations among the group included whether to refer patients with *Viannia* subgenus infection for expert otolaryngological examination regardless of symptoms (the chosen recommendation was that these patients should be referred). Another controversial topic was whether determining the *Leishmania* species responsible for the infection (requiring culture or reference laboratory support) is informative enough to make it worthwhile in most cases (again, the answer was yes). The use of serology alone (rK39) for the diagnosis of VL was discouraged, with the recommendations advocating for parasitic confirmation. The sole recommendation for which consensus was not reached was whether New World CL north of Costa Rica could be managed by observation without intervention—if healing on its own—because it is geographically thought to have less risk of metastasizing to cause ML; one member with a great deal of ML experience felt that all should receive treatment.

Preparing these leishmaniasis guidelines often led to more questions than answers. One area ripe for additional research is the management of leishmaniasis in the non-HIV immunocompromised host (e.g., post transplant or being treated with biologic immunomodulating drugs). In general, this neglected tropical disease requires fully powered sample sizes in well-done comparative clinical trials with standardized outcome measures to develop a stronger evidence base. This will likely require the collaboration of consortia across regions to refine the best treatment approaches. Until this evidence is available, the guidelines generally define simple and complicated CL as the initial decision node for local versus systemic treatment, but emphasize that treatment choice should be individualized; there is no universally applicable treatment dose, route of administration, drug, or duration of therapy.

## Conclusions

The IDSA-ASTMH clinical practice guidelines provide a sensible current approach for clinicians evaluating and treating patients with leishmaniasis. We aimed to give clear recommendations from evidence that was often inadequate, but drawing upon our collective clinical expertise in managing leishmaniasis. More leishmaniasis cases are now seen in adventurous travelers, immigrants, refugees, and military personnel. These guidelines serve as a definitive reference to assist with the management of this clinically challenging infection. Lastly, the notable absence of an effective vaccine for leishmaniasis or prophylactic treatment highlights that prevention is essential to stopping the global rise of leishmaniasis.

## Abbreviations

ASTMH: American Society of Tropical Medicine and Hygiene
CL: Cutaneous leishmaniasis
GRADE: Grading of recommendations, assessment, development, and evidence
HIV: Human immunodeficiency virus
IDSA: Infectious Diseases Society of America
ML: Mucosal leishmaniasis
VL: Visceral leishmaniasis
